# Image-Based Sexual Abuse: Characteristics Linked to Different Reasons Why Youth Decide Not to Disclose

**DOI:** 10.1007/s11121-025-01853-4

**Published:** 2025-11-14

**Authors:** Kimberly J. Mitchell, Lisa M. Jones, Ateret Gewirtz-Meydan, Jennifer O’Brien, Deirdre Colburn

**Affiliations:** 1 Crimes Against Children Research Center, University of New Hampshire, 10 West Drive, Suite 106, Durham, NH 03824 USA; 2 School of Social Work, Faculty of Social Welfare and Health Sciences, University of Haifa, Haifa, Israel; 3 The University of Texas at Arlington School of Social Work, Arlington, TX USA

**Keywords:** Nondisclosure, Image-based sexual abuse, Child sexual abuse material

## Abstract

Image-based sexual abuse is an increasingly prevalent form of technology-facilitated harm, yet disclosure rates remain low. Understanding why youth do not disclose image-based sexual abuse is critical for developing effective prevention and intervention strategies. This paper examined the reasons youth do not disclose image-based sexual abuse incidents and identified incident- and person-level characteristics associated with different nondisclosure motives. Participants were recruited online to this US-based cross-sectional study between June 28, 2023, and April 1, 2024, using social media advertisements targeting individuals aged 18–28. A total of 6204 individuals completed the survey; 2854 (46.0%) reported experiencing image-based sexual abuse before age 18. The analytic sample included 2522 incidents reported by 1551 participants that were not disclosed. The most frequently cited reasons for nondisclosure were fear of getting in trouble with family (53.9%), embarrassment (52.9%), and the belief that they could handle the incident alone (45.2%). Reasons varied by image-based sexual abuse subtype. Longer incident duration and explicit content were related to fear of getting into trouble with their family or the police, and having multiple people responsible was related to many reasons for nondisclosure (i.e., fear of getting in trouble, fear the person would find out, embarrassment, and feeling like no one could help) ,. Female participants and sexual/gender minority youth were more likely to report barriers specific to fear and shame. Prior victimization was associated with a greater belief that no one could help and fear of getting in trouble or the person finding out. Prevention should address common fears, challenge stigma and self-blame, and ensure youth have access to trusted adults and non-punitive disclosure options. These findings support clinical efforts to reduce barriers and promote safe disclosure pathways for these survivors.

## Introduction

Image-based sexual abuse (IBSA) refers to a broad set of behaviors where sexual images are created, obtained, or distributed without free and informed consent. These behaviors encompass multiple subcategories, including non-consensual taking or making of sexual images, non-consensual sharing or distribution (sometimes digitally altered), coerced or pressured production of images, threats of dissemination (“sextortion”), and commercial exploitation involving sexual images (Finkelhor et al., 2023; Henry & Beard, 2024). IBSA is increasingly recognized as a major form of technology-facilitated victimization among youth, who are especially vulnerable due to high digital engagement and peer dynamics. Behaviors that may be legal among adults (e.g., consensual image-sharing) are criminalized when minors are involved, making IBSA during adolescence both highly prevalent and uniquely consequential. Rates of IBSA victimization during adolescence are striking, yet disclosure remains notably low (Colburn et al., 2023; Joleby et al., 2024). Compared to child sexual abuse (CSA), IBSA often unfolds in digital peer and dating contexts, where the permanence and replicability of images create enduring harm and amplify barriers to telling others (Latiff et al., 2024; Manay & Collin-Vézina, 2021). This study addresses this gap by examining the reasons youth choose not to disclose IBSA experienced before the age of 18 and by identifying the incident- and person-level factors associated with nondisclosure (Pijlman et al., 2024).

IBSA is distinguished from CSA in several critical ways, primarily due to the mechanisms through which harm occurs (Pijlman et al., 2024). Whereas CSA most often involves direct contact victimization, IBSA harm is rooted in the technological permanence, replicability, and uncontrollable circulation of sexual images (Gewirtz-Meydan et al., 2018). Survivors frequently describe the abuse as ongoing, given the persistent possibility that images will resurface or be redistributed, thereby creating chronic fear and psychological distress (Canadian Centre for Child Protection, 2017; Gewirtz-Meydan et al., 2018). At the same time, important parallels make CSA a useful framework for understanding IBSA. Both forms of victimization involve violations of sexual autonomy during childhood and adolescence, and both are associated with powerful barriers to disclosure (Lemaigre et al., 2017; McPherson et al., 2025).

However, disclosure pathways also diverge: CSA is more clearly recognized within legal, clinical, and child protection frameworks, while IBSA survivors often report uncertainty about whether their experiences qualify as abuse, whether disclosure will lead to meaningful intervention, and whether they themselves will be blamed or even criminalized (Canadian Centre for Child Protection, 2017). Despite growing attention to IBSA (Colburn et al., 2023; Joleby et al., 2024), little is known about why youth choose not to disclose these experiences and what factors contribute to nondisclosure.

Given that our understanding of IBSA is still developing, existing research on CSA provides a useful starting point for informing our understanding of barriers to IBSA disclosure. In CSA, these barriers often include individual, familial, and societal factors such as internalized victim-blaming, fears of not being believed, concerns about family disruption, stigma, and limited access to supportive services (Alaggia et al., 2019; Morrison et al., 2018). IBSA survivors share many of these same challenges, yet their experiences are compounded by additional layers specific to technology-facilitated abuse.

These additional layers create unique dynamics of nondisclosure in IBSA. Whereas CSA is typically confined to specific interactions or relationships, IBSA is technology-mediated, leaving a digital trace that is permanent, replicable, and beyond the survivor’s control. This permanence amplifies fears of recognition, judgment, and stigmatization, often heightening shame and silence (ECPAT International & Fundación Renacer, 2021). IBSA may also involve blackmail or coercion (i.e., “sextortion”), where perpetrators weaponize the threat of dissemination to enforce compliance and deter disclosure (Canadian Centre for Child Protection, 2017; Martin, 2015).

Disclosure may also occur involuntarily when images are circulated without consent, possibly creating fundamentally different pathways of harm than in most CSA contexts (Gewirtz-Meydan et al., 2018). Unlike CSA, where legal pathways for reporting are more established, IBSA survivors may face uncertainty about where to seek help and whether reporting will lead to effective intervention. Additionally, survivors frequently experience profound guilt and self-blame, particularly if they initially agreed to share images that were later misused (Gewirtz-Meydan et al., 2018; Hamilton-Giachritsis et al., 2020; Schmidt et al., 2023). This internalized shame, coupled with societal victim-blaming attitudes, discourages help-seeking and limits access to support.

Beyond these interpersonal and psychological barriers, a central concern is that many youth do not disclose these experiences. Disclosure of sexual victimization in adolescence is shaped by a complex interplay of developmental and social factors (Alaggia, 2010; Augusti & Myhre, 2024; Collin-Vézina et al., 2015). Research on CSA has shown that shame, self-blame, and fear of negative reactions from caregivers and peers are major barriers to disclosure (e.g., Latiff et al., 2024). These dynamics may be amplified in the context of IBSA, where the digital permanence of images and uncertainty about their circulation create ongoing threats and heightened embarrassment (e.g., Korkman & Joleby, 2025; Paradiso et al., 2024). Peer cultures that stigmatize victims of “nudes” or frame such incidents as personal responsibility further discourage help-seeking (e.g., Burén et al., 2022; Finkelhor et al., 2023; Quayle et al., 2018; Symons et al., 2018). At the same time, adolescents’ developmental need for autonomy and fear of punitive parental responses (such as phone confiscation or restriction of internet use) may lead them to conceal incidents rather than seek support (Gemara et al., 2025). Importantly, empirical studies of IBSA survivors indicate that nondisclosure is not only common but often persists even in the face of significant distress, reflecting a perception that disclosure will not stop the harm or may even make it worse (Joleby et al., 2024)‏. Given these dynamics, examining the specific reasons why youth do not disclose IBSA is critical for understanding their coping responses and for informing prevention and intervention efforts.

These challenges are further intensified in the contemporary digital landscape of 2025. The widespread availability of encrypted messaging platforms, cloud storage, and artificial intelligence tools that enable sophisticated image manipulation (e.g., deepfake pornography) has expanded both the scale and severity of IBSA (Flynn et al., 2023). At the same time, the increasing normalization of consensual sexting among adolescents blurs boundaries between voluntary and coercive practices, adding to the uncertainty survivors feel about how to interpret their experiences. Together, these technological, cultural, and social developments underscore the importance of situating IBSA within its current context and examining the specific barriers that prevent youth from disclosing their experiences (Pijlman et al., 2024).

### The Present Study

This exploratory study surveyed a sample of young adults to retrospectively examine why youth choose not to disclose IBSA that occurred prior to age 18 and how both incident- and person-level factors are associated with these decisions. Specifically, we investigated: (1) the most common reasons participants cite for nondisclosure, (2) whether these reasons vary by IBSA type, and (3) how personal characteristics (e.g., gender, sexual minority status, victimization) and incident characteristics (e.g., relationship with person responsible, intent to harm) are associated with different reasons not to disclose.

## Methods

### Sample and Procedures

Individuals aged 18 to 28 were recruited across the USA through Facebook and Instagram advertisements between June 28, 2023, and April 1, 2024. Participants who completed the online screener and were deemed eligible were redirected to the consent and survey in Qualtrics. Two screener questions were designed to oversample individuals with IBSA experiences: sending a sexual photo or video and being asked for a sexual photo or video. Eligibility criteria were adjusted throughout recruitment to maximize the number of participants with IBSA involvement. As a result, the findings are not intended to be representative of all 18- to 28-year-olds in the USA.

The survey took an average of 30.4 min to complete. Those who completed the survey and provided a valid US non-P.O. box mailing address were sent a $15 Amazon gift card as a thank-you. Requiring a physical US mailing address for the gift card successfully deterred fraudulent entries from outside of the USA, a common challenge with online recruitment strategies that offer an email incentive (Pratt-Chapman et al., 2021).

A total of 8,596 participants completed the behavioral survey. During data cleaning, cases were dropped due to reasons such as declined consent (*n* = 227), duplicate or suspected fraud (*n* = 289), incomplete responses after consenting to participate (*n* = 1763), or having no matching screener data (*n* = 161). After appending 48 cases considered “in-progress” completed at or above 90%, we ended with a total of 6204 participants. Of these, 46.0% (*n* = 2854) reported at least one IBSA incident during childhood. These participants provided detailed information on 4205 IBSA incidents (*n* = 4149 with a response to the disclosure question). Among these, 60.8% of incidents were not disclosed by the participant, resulting in an analytic sample of 2522 undisclosed cases reported by 1,551 participants. Details of this analytic participant sample are provided in Table 1.

**Table 1:** Characteristics of participants who did not disclose image-based sexual abuse (IBSA)

Characteristic	No IBSA disclosure (*n* = 1551)
Sex assigned at birth	
Male	395 (25.5)
Female	1117 (72.0)
Intersex	14 (0.9)
Missing	25 (1.6)
Current age	
18–19	397 (25.6)
20–22	426 (27.5)
23–25	441 (28.4)
26–28	287 (18.5)
Sexual identity prior to age 18^a^	
Exclusively heterosexual	589 (38.0)
Gay/lesbian	232 (15.0)
Bisexual/queer/polysexual/demisexual	702 (45.3)
Asexual/no sexuality	44 (2.8)
Questioning/no labels	32 (2.1)
Missing	37 (2.4)
Any sexual minority identity	
No/Missing	624 (40.2)
Yes	927 (59.8)
Gender identity prior to age 18^a^	
Exclusively cisgender	1173
Transgender	139 (9.0)
Nonbinary/genderqueer/genderfluid/pangender/indigenous	272 (17.5)
Agender	39 (2.5)
Questioning/gender variant	73 (4.7)
Missing	47 (3.0)
Any gender minority identity	
No/Missing	1173 (75.6)
Yes	378 (24.4)
Race and ethnicity ^a^	
White	1163 (75.0)
Black	135 (8.7)
Asian	146 (9.4)
Hawaiian or Pacific Islander	21 (1.3)
American Indian or Alaska Native	83 (5.3)
Other non-white race	106 (6.8)
Not sure	43 (2.8)
Missing	29 (1.9)
Ethnicity	
Not Hispanic or Latino	1164 (75.1)
Hispanic or Latino	319 (20.6)
Missing	68 (4.4)
Household income	
Lower than the average family	642 (41.4)
Average	619 (39.9)
Higher than the average family	197 (12.7)
Missing	93 (6.0)
Type of community	
Small town or rural area	416 (26.8)
Suburban area next to a city	615 (39.7)
Urban or city area	465 (30.0)
Missing	55 (3.5)
Highest level of education	
Less than high school	74 (4.8)
High school diploma, GED, or trade school	411 (26.5)
Some college	442 (28.5)
Bachelor’s degree	310 ( 20.0)
Graduate school or degree	257 (16.6)
Missing	57 (3.7)
Other characteristics	
Peer norms in support of IBSA (Mean, SD)	3.48 (1.1)
Victimization (Mean, SD)	3.75 (2.0)
Non-victimization adversity (Mean, SD)	3.66 (2.3)

^a^Multiple responses possible

## Measures

### Image-based sexual abuse (IBSA)

Participants were asked questions about their experiences with five types of IBSA before age 18, using items from a scale that has undergone psychometric analyses resulting in a confirmed cohesive structure and 100% stability of all items, convergent validity, and discriminant validity (Gewirtz-Meydan et al., 2025). (1) Non-consensual taking, making, or sharing was assessed by asking whether someone had ever taken, made, or shared a sexual picture or video of them without permission, including cases where images were digitally altered. Follow-up questions determined whether the incident involved taking, making, or sharing, which were coded into two variables: (1a) taking or making and ( 1b) sharing. (2) Forced or pressured production was measured by asking whether anyone had ever threatened, pressured, or coerced them into providing sexual pictures or videos online or via a mobile device. (3) Threatened sharing was assessed by asking if someone had ever threatened to distribute a sexual image to get them ito do something they did not want to do, such as sending additional images, engaging in a sexual relationship, or providing money. (4) To capture power dynamics in IBSA, participants were asked whether, before age 18, they had ever shared sexual images or videos with someone 5 or more years older, even if they did so voluntarily. Finally, (5) commercial sexual exploitation was measured by asking whether they had ever made, sent, or posted sexual images in exchange for money, drugs, or other valuable items. Participants who endorsed at least one of these experiences were coded as having experienced IBSA.

### IBSA Incident Details

*Reasons for not disclosing.* Participants were asked whether they told anyone this was happening (yes/no). Findings on disclosure and to whom are available elsewhere (Mitchell et al., under review). For those who did not tell anyone, they were asked to mark all of the reasons why they did not tell anyone; items originated from (Wolak et al., 2006) and were further adapted and developed as part of the Technology-facilitated Abuse study (Colburn et al., 2023; Finkelhor & Turner, 2021): (1) I was afraid the person responsible would find out, (2) I was too embarrassed, (3) I thought I might get in trouble with my family, (4) I thought I might get in trouble with the police, (5) I thought I might lose access to my phone or other technology, (6) I just didn’t think to tell anyone, (7) I felt it was no big deal, (8) I didn’t think anyone could do anything to help me, and (9) I thought I could handle it on my own.

*Other incident characteristics.* Participants were asked how long the incident went on for (duration), coded as one month or longer versus less. They responded to how many times it happened, coded as four or more times versus less. The number of people responsible was queried, coded as two or more people responsible versus less. Intent to harm was measured using one follow-up item in the survey, which asked participants if the person (primarily) responsible intended to hurt, humiliate, or get back at them for something (no/yes). Whether an actual image or video was created (vs a request or threat). The explicitness of the IBSA was coded to include sexual intercourse, masturbation, naked genitals, naked breasts, or nudity without naked genitals or breasts vs not. Finally, the relationship between the participants and the person responsible was asked and coded as: stranger (vs known), adult (vs minor/unknown), whether the person responsible was a member of their family (or not), a dating partner (or not), a juvenile friend (or not), and a juvenile acquaintance (or not).

### Person-Level Factors

*Peer norms around sharing sexual material *were assessed using two questions designed to capture participants’ perceptions of their friends’ behaviors around sharing sexual pictures or videos before they turned 18; one asked about sharing with people they personally knew and the other about sharing with people they did not know personally. Both questions used a four-point Likert scale, offering response options from “none of them” to “most or all of them.” Both items were combined to create one peer norm score with acceptable reliability, especially for a 2-item scale (*α* =.71) and an average score of 3.48 (SD = 1.14) and range of 1.5–6.

*Cumulative victimization* was assessed using 8 items from the Juvenile Victimization Questionnaire (Finkelhor et al., 2005), each capturing a different type of victimization that participants may have experienced before the age of 18. Participants responded using a binary scale (0 = no, 1 = yes). These included—neglect, dating violence, witnessing assault, emotional abuse, physical abuse, witnessing domestic violence, peer exclusion, and peer name-calling. Responses were summed to create a total victimization count score ranging from 0 to 8 (M = 3.75, SD = 2.05), with higher scores reflecting greater exposure to multiple different forms of victimization.

*Lifetime non-victimization adversity* covered non-violent traumatic events and chronic stressors and was measured using 11 yes/no items: living on the street, caregiver job loss, sent or taken away from family, caregiver in prison, caregiver substance use problems, caregivers always arguing, someone close died, caregiver divorce, knew someone who overdosed, personal chronic medical condition or disability. Items were summed for a total adversity count (M = 3.66, SD = 2.25, Range: 0 to 11) (Turner & Butler, 2003).

*Sex and gender identity*. To measure sexual identity, participants were asked how they identified prior to the age of 18. Responses were combined into these categories: (1) exclusively heterosexual, (2) gay or lesbian, (3) bisexual, queer, polysexual, demisexual, (4) asexual or no sexuality, and (5) questioning or do not use labels. Gender identity was categorized as follows: (1) exclusively cisgender, (2) transgender, (3) nonbinary, genderqueer, genderfluid, pangender, indigenous, (4) agender, and (5) questioning or gender variant. For each, participants could pick all that applied to them. Additional characteristics included race, ethnicity, geographic location, income, age, and education.

## Data Analysis

 Variables with less than 5% were conservatively coded as “no;” a procedure seen across many national studies (Banyard et al., 2021; Ybarra et al., 2007). There were two exceptions were the amount of missing data was higher: the number of people responsible for the incident (7.3% missing) and the duration of the incident (15.0% missing) due to participants not being sure of the answer. For these, dummy variables were created for each to reflect a) any positive response vs all other (no, missing) and b) missing response vs all other (no, yes). These were included in multivariate analyses to account for this missing data without dropping cases.

We first compared different individual reasons for not telling anyone by type of IBSA incident using chi-square cross-tabulations overall and then conducted pairwise comparisons between IBSA types. We conducted both manual post hoc comparisons and Bonferroni adjustment with one-way ANOVA, and to be conservative, we only report those that were significant in both analyses at *p* ≤.01. Given participants could choose more than one reason for nondisclosure, multiple logistic regression models were used to assess the associations between incident- and person-level characteristics and different reasons for nondisclosure. Only non-disclosed incidents were included in analyses with comparisons between individual reasons chosen for not disclosing in comparison to incidents where that reason was not chosen.

All statistical tests were two-tailed, with the exact significance level provided. Adjusted odds ratios (aOR) and 95% confidence intervals (CI) were reported. Analyses were performed using Stata SE 19.5 (StataCorp LLC, 2025). All multivariate analyses adjusted for clustering of incidents within individual-level Response Identifiers using the Stata “cluster” command, which accounts for clustered standard errors. Multicollinearity was examined by examining correlations; the highest correlation was 0.55 (with most under 0.10), which is below the 0.8 threshold for multicollinearity.

## Results

### Reasons for Not Disclosing IBSA

As seen in Table 2, the most common reasons for not disclosing IBSA included fear of getting in trouble with family (53.9%), embarrassment (52.9%), and the belief that they could handle the situation alone (45.2%). Other frequently reported concerns included fear of losing access to technology (41.9%), the perception that no one could help (38.7%), and the belief that the incident was not a significant issue (36.9%). Some survivors indicated that they simply did not think to tell anyone (23.8%) or feared consequences involving law enforcement (20.1%). The reasons for nondisclosure varied significantly overall depending on the type of IBSA experienced. Pairwise differences between types are detailed in Table 2 with Bonferroni adjustment for multiple comparisons.

**Table 2:** Reasons for not telling anyone by type of IBSA incident

	All non-disclosed IBSA incidents (*n* = 2522)	NC taking or making (*n* = 153)	NC sharing (*n* = 277)	Threatened or forced image (*n* = 112)	Pressured image (*n* = 1080)	Threatened sharing (*n* = 206)	SP sharing with 5 + older (*n* = 652)	CSE (*n* = 42)	***X***^2^ *p* value
	*n* (%)	*n* (%)	*n* (%)	*>n* (%)	*n* (%)	*n* (%)	*n* (%)	*n* (%)	
I thought I might get in trouble with my family	1359 (53.9)	82 (53.6)	178 (64.3)	55 (49.1)	608 (56.3)	103 (50.0)	316 (48.5)^b^	17 (40.5)	<.001
I was too embarrassed	1335 (52.9)	92 (60.1)	190 (68.6)	53 (47.3)^b^	587 (54.3)^b^	112 (54.4)	285 (43.7)^a, b, d, e^	16 (38.1)^b^	<.001
I thought I could handle it on my own	1139 (45.2)	66 (43.1)	117 (42.2)	46 (41.1)	554 (51.3)	108 (52.4)	230 (35.3)^d, e^	18 (42.9)	<.001
I thought I might lose access to my phone or other technology	1057 (41.9)	42 (27.5)	122 (44.0)^a^	38 (33.9)	484 (44.8)^a^	83 (40.3)	273 (41.9)	15 (35.7)	.002
I didn’t think anyone could do anything to help me	975 (38.7)	84 (54.9)	143 (51.6)	43 (38.4)	439 (40.7)^a, b^	99 (48.1)	156 (23.9)^a, b, d, e^	11 (26.2)^a^	<.001
I felt it was no big deal	930 (36.9)	34 (22.2)	60 (21.7)	24 (21.4)	411 (38.1)^a, b, c^	41 (19.9)^d^	341 (52.3)^a, b, c, d, e^	19 (45.2)	<.001
I just didn’t think to tell anyone	601 (23.8)	39 (25.5)	49 (17.7)	23 (20.5)	269 (24.9)	36 (17.5)	176 (27.0)	9 (21.4)	.02
I thought I might get in trouble with the police	508 (20.1)	34 (22.2)	70 (25.3)	15 (13.4)	186 (17.2)	56 (27.2)	139 (21.3)	8 (19.1)	.002
I was afraid the person responsible would find out	417 (16.5)	43 (28.1)	57 (20.6)	11 (9.8)^a^	184 (17.0)^a^	60 (29.1)^c, d^	57 (8.7)^a, b, d, e^	5 (11.9)	<.001

*NC* non-consensual, *SP* self-produced, *CSE* commercial sexual exploitation

^a^Significantly different from NC taking or making; ^b^significantly different from NC sharing; ^c^significantly different from threatened or forced image; ^d^significantly different from pressured image; ^e^significantly different from threatened sharing. We conducted both manual post hoc comparisons and bonferroni adjustment with oneway ANOVA and to be conservative, we only report those that were significant in both analyses at *p* ≤.01

### Personal and Incident Characteristics Related to Nondisclosure

Several incident-level and personal factors were significantly associated with the reasons why survivors did not disclose their IBSA experiences. Fear that the person would find out was significantly associated with incidents where the person responsible was unknown, cases involving juvenile acquaintances , and instances where the person had clear intent to harm (Table 3). Embarrassment was more commonly reported among participants assigned female at birth and those who experienced IBSA incidents involving explicit content.

**Table 3:** Personal and incident characteristics related to different reasons for not disclosing (*n* = 2446)

	Afraid person would find out	*p* value	Embarrassed	*p* value
	aOR (95% CI)		aOR (95% CI)	
Incident-level				
Person responsible				
Stranger/unknown	**0.65 (0.49, 0.86)**	**0.003**	1.00 (0.81, 1.24)	0.98
Adult	1.29 (0.91, 1.82)	0.16	1.08 (0.85, 1.37)	0.54
Relative	**2.14 (1.11, 4.11)**	**0.02**	1.14 (0.56, 2.32)	0.72
Dating partner	**1.94 (1.26, 2.98)**	**0.003**	1.15 (0.84, 1.58)	0.38
Juvenile friend	**1.97 (1.11 (3.51)**	**0.02**	1.15 (0.74, 1.78)	0.54
Juvenile acquaintance	**3.14 (1.79, 5.51)**	** < 0.001**	1.01 (0.65, 1.58)	0.95
2 or more people responsible	**1.52 (1.14, 2.04)**	**0.005**	**1.47 (1.16, 1.87)**	**0.002**
Intent to harm	**1.60 (1.22, 2.09)**	**0.001**	1.23 (0.99, 1.52)	0.06
Commercial element	1.07 (0.77, 1.48)	0.70	0.86 (0.67, 1.12)	0.27
Lasted one month or more	1.21 (0.92, 1.60)	0.17	0.97 (0.79, 1.18)	0.76
Happened 4 or more times as part of same incident	**1.32 (1.02, 1.71)**	**0.03**	1.16 (0.96, 1.39)	0.12
Any IBSA image or video involved	0.77 (0.51, 1.16)	0.21	1.17 (0.88, 1.54)	0.28
Explicit image or video	1.24 (0.90, 1.72)	0.19	1.04 (0.84, 1.30)	0.70
Person-level				
Peer norms in support of IBSA	1.10 (0.92, 1.30)	0.29	0.99 (0.87, 1.12)	0.87
Victimization	**1.08 (1.00, 1.16)**	**0.04**	0.98 (0.93, 1.03)	0.40
Non-victimization adversity	1.03 (0.97, 1.09)	0.37	1.02 (0.97, 1.07)	0.48
Sexual minority identity	1.10 (0.82, 1.48)	0.52	1.02 (0.83, 1.25)	0.84
Gender minority identity	**1.42 (1.07, 1.89)**	**0.02**	0.95 (0.76, 1.19)	0.65
Female at assigned birth	**1.61 (1.17, 2.21)**	**0.003**	**1.60 (1.28, 1.99)**	** < 0.001**
Racial or ethnic minority	0.80 (0.62, 1.03)	0.08	0.93 (0.78, 1.12)	0.46
Low income	1.03 (0.81, 1.31)	0.82	0.96 (0.80, 1.16)	0.69
No high school diploma	1.04 (0.77, 1.41)	0.79	**0.73 (0.58, 0.92)**	**0.008**

Models also adjust for missing data on the number of people responsible and duration of the incident. aOR = adjusted odds ratio. Findings significant at *p* ≤.05 or better are bolded.

Fear of family consequences was particularly pronounced among incidents that lasted one month or longer, happened four or more times, and involved sexually explicit content (Table 4). This fear was also elevated in cases where the youth was female at birth and had a history of victimization. Concerns about police involvement had similar incident characteristics as fear of family consequences, but youth characteristics varied. Here, we saw increased odds of fear of police for gender minority youth and youth who reported more peer norms in support of IBSA. Fear of losing their phone or technology access was more commonly reported when the person responsible was a stranger and the incident happened four or more times. Youth with a history of victimization, who identified as sexual or gender minorities, and were assigned female at birth were also more likely to report this barrier to disclosure. Racial or ethnic minority youth were more likely to say they did not think of telling anyone about the IBSA (Table 5). Feeling like no one could help was more common when there were multiple people responsible, there was intent to harm, among youth with a victimization history, and among those who were female.

**Table 4:** Personal and incident characteristics related to different reasons for not disclosing (*n* = 2446)

	Afraid would get in trouble with family		Afraid would get in trouble with police		Afraid phone would be taken away	
	aOR (95% CI)	*p* value	aOR (95% CI)	*p* value	aOR (95% CI)	*p* value
Incident-level						
Person responsible						
Stranger	1.15 (0.92, 1.43)	0.23	1.12 (0.86, 1.47)	0.39	**1.53 (1.22, 1.91)**	** < 0.001**
Adult	1.19 (0.93, 1.52)	0.16	0.99 (0.74, 1.34)	0.97	**0.69 (0.54, 0.88)**	**0.003**
Relative	1.67 (0.79, 3.53)	0.18	1.55 (0.82, 2.91)	0.17	1.26 (0.61, 2.59)	0.53
Dating partner	1.07 (0.77, 1.48)	0.68	0.69 (0.45, 1.06)	0.09	**0.63 (0.45, 0.88)**	**0.006**
Juvenile friend	1.09 (0.72, 1.67)	0.67	0.60 (0.31, 1.14)	0.12	**0.52 (0.33, 0.82)**	**0.005**
Juvenile acquaintance	0.84 (0.53, 1.32)	0.44	0.94 (0.51, 1.71)	0.83	0.70 (0.43, 1.14)	0.15
2 or more people responsible	**1.36 (1.06, 1.74)**	**0.02**	**1.45 (1.07, 1.95)**	**0.01**	1.23 (0.95, 1.57)	0.11
Intent to harm	**1.32 (1.05, 1.65)**	**0.02**	**1.33 (1.03, 1.72)**	**0.03**	0.83 (0.66, 1.05)	0.12
Commercial element	**0.70 (0.52, 0.93)**	**0.01**	1.26 (0.92, 1.71)	0.15	0.78 (0.59, 1.04)	0.09
Lasted one month or more	**1.50 (1.21, 1.86)**	** < 0.001**	**1.28 (0.99, 1.66)**	**0.05**	1.11 (0.90, 1.38)	0.32
Happened 4 or more times as part of same incident	**1.25 (1.03, 1.51)**	**0.02**	1.16 (0.92, 1.45)	0.21	**1.35 (1.12, 1.63)**	**0.001**
Any IBSA image or video involved	0.88 (0.66, 1.16)	0.37	1.33 (0.86, 2.04)	0.20	**0.67 (0.50, 0.89)**	**0.006**
Sexually explicit image or video	**1.31 (1.05, 1.65)**	**0.02**	**1.38 (1.02, 1.86)**	**0.04**	1.25 (0.99, 1.58)	0.07
Person-level						
Peer norms in support of IBSA	**0.84 (0.74, 0.96)**	**0.01**	**1.19 (1.01, 1.39)**	**0.04**	1.04 (0.91, 1.19)	0.59
Victimization	**1.06 (1.00, 1.12)**	**0.04**	1.01 (0.95, 1.09)	0.69	**1.07 (1.01, 1.13)**	**0.02**
Non-victimization adversity	0.99 (0.94, 1.04)	0.65	1.05 (0.99, 1.12)	0.12	0.99 (0.94, 1.05)	0.82
Sexual minority identity	1.13 (0.92, 1.40)	0.24	1.22 (0.93, 1.62)	0.15	**1.33 (1.07, 1.66)**	**0.01**
Gender minority identity	**1.28 (1.01, 1.62)**	**0.04**	**1.45 (1.12, 1.89)**	**0.005**	**1.30 (1.03, 1.64)**	**0.03**
Female at assigned birth	**2.29 (1.83, 2.87)**	** < 0.001**	1.13 (0.86, 1.49)	0.38	**1.74 (1.37, 2.20)**	** < 0.001**
Racial or ethnic minority	0.92 (0.76, 1.11)	0.38	**0.79 (0.62, 0.99)**	**0.05**	0.86 (0.71, 1.05)	0.15
Low income	1.05 (0.87, 1.28)	0.59	1.01 (0.80, 1.27)	0.94	0.95 (0.78, 1.16)	0.62
No high school diploma	0.86 (0.68, 1.09)	0.22	**0.61 (0.45, 0.84)**	**0.002**	0.97 (0.76, 1.25)	0.85

Models also adjust for missing data on the number of people responsible and duration of the incident. *aOR* adjusted odds ratio. Findings significant at *p* ≤.05 or better are bolded

**Table 5:** Personal and incident characteristics related to different reasons for not disclosing (*n* = 2,446)

	**Didn’t think to tell anyone**		**Thought is was not a big deal**		**Felt no one could help**		**Thought could handle it on own**	
	**aOR (95% CI)**	*P* value	**aOR (95% CI)**	*P* value	**aOR (95% CI)**	*P* value	**aOR (95% CI)**	*P* value
Incident-level								
Person responsible								
Stranger	1.03 (0.81, 1.32)	0.78	1.18 (0.95, 1.46)	0.13	**0.80 (0.64, 1.00)**	**0.05**	**0.76 (0.61, 0.94)**	**0.01**
Adult	0.77 (0.59, 1.01)	0.06	0.87 (0.69, 1.11)	0.28	0.92 (0.72, 1.17)	0.50	0.86 (0.67, 1.09)	0.21
Relative	0.46 (0.18, 1.14)	0.09	0.75 (0.32, 1.73)	0.50	1.48 (0.70, 3.14)	0.30	1.06 (0.54, 2.09)	0.87
Dating partner	0.85 (0.59, 1.22)	0.37	0.91 (0.66, 1.25)	0.56	0.89 (0.65, 1.24)	0.50	**0.68 (0.49, 0.94)**	**0.02**
Juvenile friend	1.08 (0.65, 1.79)	0.76	1.03 (0.65, 1.64)	0.88	0.95 (0.60, 1.51)	0.83	0.89 (0.56, 1.41)	0.62
Juvenile acquaintance	0.85 (0.51, 1.42)	0.54	0.73 (0.45, 1.19)	0.21	0.68 (0.42, 1.10)	0.12	0.85 (0.55, 1.33)	0.48
2 or more people responsible	0.91 (0.68, 1.21)	0.52	**0.71 (0.55, 0.92)**	**0.01**	**1.97 (1.55, 2.50)**	**<0.001**	1.15 (0.91, 1.46)	0.25
Intent to harm	**0.75 (0.58, 0.97)**	**0.03**	**0.60 (0.48, 0.74)**	**<0.001**	**1.61 (1.30, 2.01)**	**<0.001**	0.88 (0.71, 1.10)	0.26
Commercial element	0.93 (0.69, 1.27)	0.67	0.78 (0.59, 1.03)	0.08	0.93 (0.70, 1.24)	0.64	1.03 (0.79, 1.34)	0.84
Lasted one month or more	0.86 (0.67, 1.09)	0.22	0.94 (0.77, 1.17)	0.60	1.02 (0.83, 1.26)	0.85	0.96 (0.78, 1.19)	0.73
Happened 4 or more times	0.91 (0.74, 1.13)	0.40	1.01 (0.84, 1.22)	0.89	1.10 (0.91, 1.33)	0.31	**1.30 (1.08, 1.56)**	**0.006**
Any IBSA image or video involved	1.21 (0.88, 1.65)	0.24	1.18 (0.88, 1.57)	0.26	0.75 (0.56, 1.01)	0.06	**0.54 (0.41, 0.72)**	**<0.001**
Explicit image or video	0.91 (0.71, 1.17)	0.47	1.10 (0.88, 1.39)	0.40	1.25 (0.99, 1.59)	0.06	1.03 (0.82, 1.28)	0.83
Person-level								
Peer norms in support of IBSA	0.97 (0.84, 1.13)	0.73	0.93 (0.81, 1.06)	0.29	1.09 (0.95, 1.24)	0.21	1.04 (0.91, 1.18)	0.57
Victimization count	0.97 (0.91, 1.03)	0.30	**0.94 (0.89, 0.99)**	**0.03**	**1.10 (1.04, 1.16)**	**0.001**	1.03 (0.97, 1.08)	0.34
Non-victimization adversity	**0.94 (0.89, 1.00)**	**0.04**	1.01 (0.96, 1.06)	0.75	1.00 (0.95, 1.05)	0.90	1.00 (0.95, 1.05)	0.89
Sexual minority identity	1.22 (0.97, 1.54)	0.09	1.17 (0.95, 1.45)	0.13	1.12 (0.90, 1.39)	0.30	1.01 (0.83, 1.24)	0.91
Gender minority identity	0.83 (0.63, 1.08)	0.16	1.02 (0.81, 1.28)	0.87	1.19 (0.95, 1.50)	0.13	1.06 (0.84, 1.32)	0.63
Female at assigned birth	0.86 (0.67, 1.09)	0.21	**0.76 (0.61, 0.94)**	**0.01**	**1.37 (1.10, 1.72)**	**0.005**	1.21 (0.98, 1.49)	0.08
Racial or ethnic minority	**1.43 (1.16, 1.76)**	**0.001**	1.02 (0.85, 1.23)	0.79	**0.75 (0.62, 0.91)**	**0.004**	0.99 (0.82, 1.19)	0.91
Low income	1.06 (0.86, 1.32)	0.57	1.11 (0.92, 1.35)	0.26	1.03 (0.85, 1.24)	0.78	1.07 (0.89, 1.29)	0.48
No high school diploma	0.89 (0.68, 1.17)	0.41	0.97 (0.76, 1.24)	0.81	0.83 (0.65, 1.07)	0.14	**0.76 (0.59, 0.97)**	**0.03**

Models also adjust for missing data on the number of people responsible and duration of the incident. *aOR* adjusted odds ratio. Findings significant at *p* <.05 or better are bolded

## Discussion

This study examined the reasons why youth who experience IBSA choose not to disclose these incidents, highlighting both incident-level and personal characteristics that shape their silence. Four domains emerged as central to nondisclosure: (1) fear, (2) embarrassment, (3) perceptions of self-sufficiency or minimization, and (4) perceived futility of disclosure. These findings suggest that nondisclosure of IBSA reflects barriers well documented in the CSA literature while also revealing additional dynamics unique to technology-facilitated abuse. Understanding both the overlap and divergence between IBSA and CSA is critical for developing prevention strategies that address the distinctive risks posed by digitally mediated forms of sexual abuse.

Fear emerged as a pervasive barrier to disclosure, echoing CSA literature that documents how anticipated family conflict or punishment often deters disclosure (Alaggia et al., 2019; Pijlman et al., 2024; Sparks, 2022). Participants in our sample commonly feared being blamed, punished, or having their devices taken away, particularly in incidents involving repeated victimization or sexually explicit content. This indicates that prevention messages emphasizing device restriction or punitive responses may inadvertently heighten silence rather than encourage disclosure. Programs must therefore focus on non-punitive strategies that emphasize safety and support rather than punishment (Henry et al., 2018).

Fear also extended to law enforcement involvement. Consistent with prior research on CSA and technology-facilitated abuse, participants anticipated that authorities might dismiss their experiences as consensual or fail to act effectively, especially when images were initially shared voluntarily (Canadian Centre for Child Protection, 2017; Flynn et al., 2023; Gewirtz-Meydan et al., 2018; O’Brien et al., 2025). This highlights a crucial prevention implication: unless authorities are trained to recognize IBSA as abuse and respond in non-blaming ways, survivors will remain reluctant to disclose (Huber, 2025).

Finally, fear of retaliation was especially pronounced in more severe IBSA incidents (e.g., multiple perpetrators, sexually explicit images, clear intent to harm). This aligns with findings in CSA disclosure research that suggests proximity to the perpetrator complicates help-seeking (Latiff et al., 2024), but the digital nature of IBSA amplifies risks of continued harassment or wider image circulation. Prevention must therefore include measures that address retaliation fears, including safe, anonymous reporting channels.

Embarrassment was another prevalent barrier, particularly for female participants and incidents with more explicit content. Shame and stigma have long been documented as central to CSA nondisclosure (Alaggia et al., 2019; Kennedy & Prock, 2018; Sivagurunathan et al., 2019), but in IBSA, this embarrassment is often magnified by cultural narratives that conflate consensual image sharing with moral failure (Flynn et al., 2023; Umbach et al., 2025). The visibility and permanence of digital images may heighten these feelings, especially when peers or communities uphold punitive or judgmental norms (Canadian Centre for Child Protection, 2017; Gewirtz-Meydan et al., 2018; Nygård et al., 2024). This underscores the importance of prevention efforts that challenge victim-blaming norms and provide youth with affirming, non-judgmental spaces for disclosure (Flynn et al., 2023).

Perceived self-sufficiency—sometimes described in this study as “minimization” (participants’ beliefs that the incident was not serious or harmful enough to warrant telling someone, which is closely related to, but distinct from, normalization)—was another major barrier to disclosing IBSA. This included youth who believed they could handle the incident on their own, did not view it as serious, or simply did not think to tell anyone (Pijlman, 2025; Pijlman et al., 2025). These perceptions may stem from normalization of digital sexual exchanges among adolescents, especially in peer environments where sexting is widespread and IBSA may not be clearly distinguished as abusive (Henry & Beard, 2024). In some cases, the lack of disclosure may reflect resilience or a genuine assessment of low harm; in others, it may indicate unawareness of long-term risks or internalized blame (Powell et al., 2022). Notably, this cluster of reasons was more common in cases involving self-produced image sharing with older individuals or where coercion was subtle rather than overt—raising concerns about grooming dynamics and power imbalances that are not necessarily recognized by the youth as exploitative. Prevention initiatives should therefore focus on increasing digital literacy and helping youth recognize exploitative dynamics even when coercion is indirect.

Finally, many youth reported a belief that “no one could help,” reflecting systemic failures in support systems (Umbach et al., 2025). This belief can be particularly pronounced in more severe cases, including incidents with multiple people responsible, clear intent to harm, or prolonged abuse (McElvaney, 2015). Such experiences may foster learned helplessness, especially among youth with histories of prior victimization or failed disclosure attempts (Alaggia et al., 2019). The association between cumulative victimization and this perception in the current analyses further underscores how IBSA often occurs in the context of complex trauma and structural vulnerability (Mitchell et al., 2025a, 2025b). This sense of futility may also correspond with broader patterns of victim-blaming specific to IBSA, where youth anticipate being held responsible for their own victimization, particularly when images were initially shared voluntarily, rather than being seen as legitimate victims (Powell et al., 2022; Sparks, 2022). This highlights the importance of systemic change: youth must not only be encouraged to disclose but must also encounter consistent, effective, and non-blaming responses when they do.

Importantly, the type of IBSA experienced shaped the reasons for nondisclosure. Survivors of non-consensual image sharing most often feared family punishment or embarrassment, while those subjected to image threats (e.g., sextortion) often cited fear of retaliation. These patterns reflect the different psychological and social consequences tied to each subtype and suggest that a one-size-fits-all prevention approach is insufficient. Tailored messaging that acknowledges the specific fears associated with each form of IBSA may better support disclosure and recovery.

The study also highlighted the roles of relationships with the people responsible and personal identity characteristics. Responsible parties who were dating partners, relatives, or peers were associated with higher rates of fear-based nondisclosure barriers. This finding parallels CSA research showing that closeness to the perpetrator intensifies internal conflict and complicates help-seeking (Latiff et al., 2024). Moreover, SGM youth were more likely to cite fear of familial and police consequences, echoing literature on minority stress and the compounded vulnerabilities of LGBTQ + youth in disclosure contexts (Meechan-Rogers et al., 2021; Turner et al., 2024; Umbach et al., 2025). These findings suggest that IBSA disclosure is shaped not only by the digital environment but also by the same relational and identity-based dynamics that characterize offline forms of sexual victimization. Indeed, results suggest that online and offline contexts often parallel one another: patterns of nondisclosure and anticipated consequences in digital abuse reflect similar processes observed in face-to-face abuse. Prevention and intervention efforts should therefore be designed to bridge these contexts rather than treating them as wholly separate. At the same time, important distinctions remain. Digital abuse involves features such as image permanence, replicability, and viral circulation, which can create forms of harm and deterrents to disclosure that differ from those found in offline settings. Recognizing both the overlaps and divergences between online and offline abuse is therefore critical to tailoring effective responses.

### Limitations

Several limitations warrant consideration. First, the study relied on retrospective self-reports from young adults (ages 18–28) about IBSA that occurred before age 18. While retrospective designs are common in sexual abuse research, recall bias and reinterpretation of events over time may have influenced responses. Second, because the survey was conducted online and relied on voluntary participation, the sample may overrepresent individuals who are comfortable engaging in web-based surveys. 

Third, while the survey included both incident-level and person-level measures, disclosure decisions are complex, context-dependent, and often unfold over time. Our cross-sectional design does not capture the temporal sequencing of experiences or the ways disclosure may evolve following changes in relationships, technology use, or institutional responses. Moreover, disclosure is not always binary; youth may disclose partially, indirectly, or to some but not others. These nuances could not be assessed within the current quantitative framework. Fourth, although we examined key personal factors such as peer norms, cumulative victimization, and minority identity, other potentially relevant influences, such as family communication styles, community cultural norms, or prior experiences with institutional authorities, were not measured and should be incorporated into future research.

Finally, the study was not intended to produce nationally representative prevalence estimates. Rather, the sample reflects a large and diverse group of young adults recruited online, providing important insights into patterns of IBSA nondisclosure but not generalizable rates. Future research employing mixed-methods, longitudinal designs, and samples that intentionally include underrepresented populations will be essential for deepening our understanding of the mechanisms underlying disclosure and nondisclosure.

### Future Research Directions

In line with a resilience-oriented perspective, future research should not only identify barriers but also examine the circumstances under which disclosure becomes possible. Studies could focus on youth who disclose and analyze the protective factors that support this process (e.g., supportive peers, trusted adults, or positive institutional responses). Such work would highlight protective pathways and inform strategies that foster safe disclosure environments.

Future investigations would also benefit from approaches that capture the *processual* nature of disclosure. Because disclosure is rarely a single event but often unfolds gradually or selectively, prospective, developmental, and mixed-methods designs could illuminate how decisions shift over time, across relationships, and in response to cumulative experiences of victimization. In addition, qualitative and participatory research is needed to deepen understanding of the emotional, relational, and cultural dynamics that shape disclosure, especially among youth with marginalized identities whose perspectives are often overlooked.

Finally, as digital environments evolve, research should explore how new technologies, including encrypted messaging, ephemeral platforms, and AI-driven image manipulation, affect both the risks of IBSA and the possibilities for disclosure. Attending to these technological shifts alongside offline relational and cultural contexts will be crucial for designing interventions that remain relevant to youth realities.

## Conclusion

The findings highlight the unique and multifaceted reasons why youth choose not to disclose IBSA. Fear, shame, perceived futility, and minimization each play critical roles, and these are shaped by both the nature of the abuse and the identities and life circumstances of the survivors. Prevention and intervention efforts must shift toward affirming, context-sensitive frameworks that empower youth to seek support while minimizing shame and fear. Building youth trust in systems, and increasing access to non-judgmental support, is essential to reducing the silence that continues to surround IBSA.

## Data Availability

Data is available from the corresponding author upon reasonable request.

## References

[CR1] Alaggia, R. (2010). An ecological analysis of child sexual abuse disclosure: Considerations forchild and adolescent mental health. *Journal of the Canadian Academy of Child and Adolescent Psychiatry,**19*(1), 32. 20119565 PMC2809444

[CR2] Alaggia, R., Collin-Vézina, D., & Lateef, R. (2019). Facilitators and barriers to child sexual abuse (CSA) disclosures: A research update (2000–2016). *Trauma, Violence & Abuse,**20*(2), 260–283. 10.1177/1524838017697312 PMC6429637 29333973

[CR3] Augusti, E. M., & Myhre, M. C. (2024). The barriers and facilitators to abuse disclosure andpsychosocial support needs in children and adolescents around the time ofdisclosure. *Child Care in Practice,**30*(2), 187–202.

[CR4] Banyard, V., Mitchell, K. J., & Ybarra, M. L. (2021). Exposure to self-directed violence: Understanding intention to help and helping behaviors among adolescents and emerging adults. *International Journal of Environmental Research and Public Health,**18*(16), Article 8606. 34444354 10.3390/ijerph18168606 PMC8391527

[CR5] Burén, J., Holmqvist Gattario, K., & Lunde, C. (2022). What do peers think about sexting? Adolescents’ views of the norms guiding sexting behavior. *Journal of Adolescent Research,**37*(2), 221–249.

[CR6] Canadian Centre for Child Protection. (2017). *Survivors*’ *survey: Full report 2017*. https://content.c3p.ca/pdfs/C3P_SurvivorsSurveyFullReport2017.pdf. Accessed 20 Oct 2025.

[CR7] Colburn, D. A., Finkelhor, D., & Turner, H. A. (2023). Help-seeking from websites and police in the aftermath of technology-facilitated victimization. *Journal of Interpersonal Violence,**38*(21–22), 11642–11665. 37458155 10.1177/08862605231186156

[CR8] Collin-Vézina, D., De La Sablonnière-Griffin, M., Palmer, A. M., & Milne, L. (2015). Apreliminary mapping of individual, relational, and social factors that impede disclosure ofchildhood sexual abuse. *Child Abuse & Neglect,**43*, 123–134. 25846196 10.1016/j.chiabu.2015.03.010

[CR9] ECPAT International, & Fundación Renacer. (2021). *Child sexual exploitation and abuse online: Survivors*’ *perspectives in Colombia*. ECPAT International. https://ecpat.org/wp-content/uploads/2021/12/30-11-2021_Colombia_National-Report_EN_FINAL.pdf. Accessed 20 Oct 2025.

[CR10] Finkelhor, D., Hamby, S. L., Ormrod, R., & Turner, H. (2005). The juvenile victimization questionnaire: Reliability, validity, and national norms. *Child Abuse & Neglect,**29*(4), 383–412. 15917079 10.1016/j.chiabu.2004.11.001

[CR11] Finkelhor, D., & Turner, H. (2021). *Victims of technology-facilitated abuse: Prevalence, awareness, dynamics, help-seeking and reporting, United States, 2021*. Inter-university Consortium for Political and Social Research. https://www.icpsr.umich.edu/web/ICPSR/studies/38917. Accessed 6 Oct 2025.

[CR12] Finkelhor, D., Turner, H., Colburn, D., Mitchell, K., & Mathews, B. (2023). Child sexual abuse images and youth produced images: The varieties of image-based sexual exploitation and abuse of children. *Child Abuse & Neglect,**143*, Article 106269. 37336088 10.1016/j.chiabu.2023.106269

[CR13] Flynn, A., Cama, E., Powell, A., & Scott, A. J. (2023). Victim-blaming and image-based sexual abuse. *Journal of Criminology,**56*(1), 7–25.

[CR14] Gemara, N., Mishna, F., & Katz, C. (2025). ‘If my parents find out, I will not see my phone anymore’: Who do children choose to disclose online sexual solicitation to? *Child & Family Social Work,**30*(1), 4–14.

[CR15] Gewirtz-Meydan, A., Turner, H. A., Finkelhor, D., Jones, L. M., Colburn, D. A., & Mitchell, K. J. (2025). Measuring image-based sexual abuse (IBSA): Psychometric validation and analysis of the IBSA Scale. *Child Maltreatment*, 10775595251338188. 10.1177/10775595251338188 10.1177/10775595251338188 PMC12992646 40328687

[CR16] Gewirtz-Meydan, A., Walsh, W., Wolak, J., & Finkelhor, D. (2018). The complex experience of child pornography survivors. *Child Abuse & Neglect,**80*, 238–248. 29631255 10.1016/j.chiabu.2018.03.031

[CR17] Hamilton-Giachritsis, C., Hanson, E., Whittle, H., Alves-Costa, F., & Beech, A. (2020). Technology assisted child sexual abuse in the UK: Young people’s views on the impact of online sexual abuse. *Children and Youth Services Review,**119*, 105451.

[CR18] Henry, N., & Beard, G. (2024). Image-based sexual abuse perpetration: A scoping review. *Trauma, Violence, & Abuse,**25*(5), 3981–3998. 10.1177/15248380241266137 PMC11545137 39078000

[CR19] Henry, N., Flynn, A., & Powell, A. (2018). Policing image-based sexual abuse: Stakeholder perspectives. *Police Practice and Research,**19*(6), 565–581.

[CR20] Huber, A. R. (2025). Image-based sexual abuse: Legislative and policing responses. *Criminology & Criminal Justice,**25*(3), 736–752.

[CR21] Joleby, M., Landström, S., Lunde, C., & Jonsson, L. S. (2024). “But I wanted to talk about it”: Technology-assisted child sexual abuse victims’ reasoning for delayed disclosure. *Child Protection and Practice,**3*, Article 100062.

[CR22] Kennedy, A. C., & Prock, K. A. (2018). “I still feel like I am not normal”: A review of the role of stigma and stigmatization among female survivors of child sexual abuse, sexual assault, and intimate partner violence. *Trauma, Violence, & Abuse,**19*(5), 512–527. 10.1177/1524838016673601 27803311

[CR23] Korkman, J., & Joleby, M. (2025). Disclosures and forensic interviews in the context of online child sexual abuse. In M. E. Lamb, I. Hershkowitz & M. E. Pipe (Eds.), *Child Sexual Abuse: Why Children Disclose or Deny Being Abused* (2nd ed., pp. 150–196). Routledge.

[CR24] Latiff, M. A., Fang, L., Goh, D. A., & Tan, L. J. (2024). A systematic review of factors associated with disclosure of child sexual abuse. *Child Abuse & Neglect,**147*, Article 106564. 38056036 10.1016/j.chiabu.2023.106564

[CR25] Lemaigre, C., Taylor, E. P., & Gittoes, C. (2017). Barriers and facilitators to disclosing sexual abuse in childhood and adolescence: A systematic review. *Child Abuse & Neglect,**70*, 39–52. 28551460 10.1016/j.chiabu.2017.05.009

[CR26] Manay, N., & Collin-Vézina, D. (2021). Recipients of children’s and adolescents’ disclosures of childhood sexual abuse: A systematic review. *Child Abuse & Neglect,**116*, 104192. 31564382 10.1016/j.chiabu.2019.104192

[CR27] Martin, J. (2015). Conceptualizing the harms done to children made the subjects of sexual abuse images online. *Child & Youth Services,**36*(4), 267–287.

[CR28] McElvaney, R. (2015). Disclosure of child sexual abuse: Delays, non-disclosure and partial disclosure. What the research tells us and implications for practice. *Child Abuse Review,**24*(3), 159–169.

[CR29] McPherson, L., Gatwiri, K., Graham, A., Rotumah, D., Hand, K., Modderman, C., Chubb, J., & James, S. (2025). What helps children and young people to disclose their experience of sexual abuse and what gets in the way? A systematic scoping review. *Child & Youth Care Forum,**54*(2), 515–544.

[CR30] Meechan-Rogers, R., Jones, C. B., & Ward, N. (2021). Image-based sexual abuse: An LGBTQ+ perspective. In *The Palgrave handbook of gendered violence and technology* (pp. 297–318).

[CR31] Mitchell, K. J., Colburn, D., Finkelhor, D., Gewirtz-Meydan, A., Turner, H. A., & Jones, L. M. (2025). Links between image-based sexual abuse and mental health in childhood among young adult social media users. *Child Abuse & Neglect,**164*, Article 107471. 40273655 10.1016/j.chiabu.2025.107471

[CR32] Mitchell, K., Colburn, D., Gewirtz-Meydan, A., Finkelhor, D., Jones, L., Turner, H., & O'Brien, J. (under review). Childhood image-based sexual abuse: Recipients of disclosure from a diverse group of young adults. *Child Protection and Practice*.

[CR33] Mitchell, K. J., Gewirtz-Meydan, A., Jones, L. M., & Turner, H. A. (2025). Image-based sexual abuse profiles: Integrating mental health, adversities, and victimization to explore social contexts in a diverse group of young adults. *Computers in Human Behavior*. 10.1016/j.chb.2025.108717

[CR34] Morrison, S. E., Bruce, C., & Wilson, S. (2018). Children’s disclosure of sexual abuse: A systematic review of qualitative research exploring barriers and facilitators. *Journal of Child Sexual Abuse,**27*(2), 176–194. 29488844 10.1080/10538712.2018.1425943

[CR35] Nygård, S., Kvalem, I. L., & Træen, B. (2024). “It spread like wildfire, as these things do”: Exploring mechanisms of harm in young Norwegians’ experiences of image-based sexual abuse. *The Journal of Sex Research*, 1–18. 10.1080/00224499.2024.2341129 38629688

[CR36] O’Brien, J. E., Kahn, G. Z., Gast, L., & Mitchell, K. J. (2025). “They are not victimless crimes… that’s frustrating to hear”: Qualitative insights from prosecutors working on cases related to technology facilitated child sexual abuse material. *Child Abuse & Neglect,**159*, Article 107169. 39631178 10.1016/j.chiabu.2024.107169

[CR37] Paradiso, M. N., Rollè, L., & Trombetta, T. (2024). Image-based sexual abuse associated factors: A systematic review. *Journal of Family Violence,**39*(5), 931–954. 10.1007/s10896-023-00557-z PMC10126554 37358981

[CR38] Pijlman, V. E. (2025). *Caught in the web: A study of help-seeking for sexual victimization* (Publication Number 9789465069845) Vrije Universiteit Amsterdam].

[CR39] Pijlman, V., Eichelsheim, V., Pemberton, A., & Waardt, Md. (2025). “I did not want to make a bigger deal out of it than it was”: A mixed-method study on the help-seeking behavior of victims of image-based sexual harassment and abuse. *Journal of Interpersonal Violence,**40*(5–6), 1325–1359. 38910535 10.1177/08862605241258996

[CR40] Pijlman, V., Waardt, M. d., Schoonmade, L., Eichelsheim, V., & Pemberton, A. (2024). The help-seeking behavior of victims of image-based sexual harassment and abuse: A scoping review. *Trauma, Violence, & Abuse*, 15248380241289435. 10.1177/15248380241289435 39427323

[CR41] Powell, A., Scott, A. J., Flynn, A., & McCook, S. (2022). Perpetration of image-based sexual abuse: Extent, nature and correlates in a multi-country sample. *Journal of Interpersonal Violence,**37*(23–24), NP22864–NP22889. 35184577 10.1177/08862605211072266

[CR42] Pratt-Chapman, M., Moses, J., & Arem, H. (2021). Strategies for the identification and prevention of survey fraud: Data analysis of a web-based survey. *JMIR Cancer,**7*(3), Article e30730. 34269685 10.2196/30730 PMC8325077

[CR43] Quayle, E., Jonsson, L. S., Cooper, K., Traynor, J., & Svedin, C. G. (2018). Children in identified sexual images–who are they? Self-and non-self-taken images in the International Child Sexual Exploitation Image Database 2006–2015. *Child Abuse Review,**27*(3), 223–238.

[CR44] Schmidt, F., Varese, F., & Bucci, S. (2023). Understanding the prolonged impact of online sexual abuse occurring in childhood. *Frontiers in Psychology,**14*, Article 1281996. 37941760 10.3389/fpsyg.2023.1281996 PMC10627921

[CR45] Sivagurunathan, M., Orchard, T., MacDermid, J. C., & Evans, M. (2019). Barriers and facilitators affecting self-disclosure among male survivors of child sexual abuse: The service providers’ perspective. *Child Abuse & Neglect,**88*, 455–465. 30219431 10.1016/j.chiabu.2018.08.015

[CR46] Sparks, B. (2022). A snapshot of image-based sexual abuse (IBSA): Narrating a way forward. *Sexuality Research & Social Policy,**19*(2), 689–704. 33936320 10.1007/s13178-021-00585-8 PMC8076670

[CR47] StataCorp LLC. (2025). *StataNow/SE 19.5* In https://www.stata.com/. Accessed 20 Oct 2025.

[CR48] Symons, K., Ponnet, K., Walrave, M., & Heirman, W. (2018). Sexting scripts in adolescent relationships: Is sexting becoming the norm? *New Media & Society,**20*(10), 3836–3857.

[CR49] Turner, H. A., & Butler, M. J. (2003). Direct and indirect effects of childhood adversity on depressive symptoms in young adults. *Journal of Youth and Adolescence,**32*(2), 89–103.

[CR50] Turner, H. A., Finkelhor, D., Mitchell, K., & Colburn, D. (2024). Prevalence of technology-facilitated abuse among sexual and gender minority youths. *JAMA Network Open,**7*(2), e2354485–e2354485. 38306097 10.1001/jamanetworkopen.2023.54485 PMC10837746

[CR51] Umbach, R., Henry, N., & Beard, G. (2025). Prevalence and impacts of image-based sexual abuse victimization: A multinational study. *arXiv preprint * arXiv:2503.04988 .

[CR52] Wolak, J., Mitchell, K. J., & Finkelhor, D. (2006). *Online Victimization of Youth: Five Years Later*.

[CR53] Ybarra, M. L., Espelage, D. L., & Mitchell, K. J. (2007). The co-occurrence of Internet harassment and unwanted sexual solicitation victimization and perpetration: Associations with psychosocial indicators. *Journal of Adolescent Health,**41*(6 Suppl 1), S31-41. 10.1016/j.jadohealth.2007.09.010 10.1016/j.jadohealth.2007.09.010 18047943

